# Whole Blood Levels of the n-6 Essential Fatty Acid Linoleic Acid Are Inversely Associated with Stunting in 2-to-6 Year Old Tanzanian Children: A Cross-Sectional Study

**DOI:** 10.1371/journal.pone.0154715

**Published:** 2016-05-03

**Authors:** Theresia Jumbe, Sarah S. Comstock, Samantha L. Hahn, William S. Harris, Joyce Kinabo, Jenifer I. Fenton

**Affiliations:** 1 Department of Food Science and Human Nutrition, Michigan State University, East Lansing, Michigan, United States of America; 2 Sanford School of Medicine, University of South Dakota and OmegaQuant Analytics, LLC, Sioux Falls, South Dakota, United States of America; 3 Sokoine University of Agriculture, Morogoro, Tanzania; Laurentian University, CANADA

## Abstract

**Background:**

In Tanzania, 35% of all children below five years of age are stunted. Dietary fatty acids (FA) are critical for growth and development. However, whole blood FA levels in Tanzanian children are poorly described.

**Objective:**

The objectives of this cross-sectional study were to assess 1) whole blood levels of essential fatty acids and 2) the association between whole blood FA levels and growth parameters in Tanzanian children 2–6 years of age.

**Methods:**

A drop of blood was collected on an antioxidant treated card and analyzed for FA composition. Weight and height were measured and *z*-scores calculated. Relationships between FAs and growth parameters were analyzed by linear regression.

**Results:**

Of the 334 children that participated, 30.3% were stunted. The average whole blood level of Mead acid was 0.15%. The anthropometric *z*-score height-for-age (HAZ) was inversely associated with Mead acid, the Mead acid to arachidonic acid (T/T) ratio, and total n-9 FA. Additionally, HAZ was positively associated with linoleic acid and total n-6 FA. BMI-for-age was positively associated with oleic acid, total n-9 FA and T/T ratio but inversely associated with arachidonic acid and total n-6 FA. Weight-for-height was inversely associated with arachidonic acid and total n-6 FAs and positively associated with oleic acid and total n-9 FA. Weight-for-age was not associated with any FA tested. Total n-3 FAs were not associated with any growth parameters measured.

**Conclusions:**

The EFA linoleic acid and the markers of FA deficiency were associated with HAZ, an indicator for stunting in 2–6 year old Tanzanian children. Total n-6, total n-9, and a number of individual FAs were associated with growth. Increasing dietary intake of EFA and n-6 FAs may be a strategy to combat stunting in this population.

## Introduction

Stunting, an inability to achieve height growth potential, is one of the major nutritional challenges in Tanzania. Approximately 35% of children in Tanzania are stunted [1]. Only recently, supplementation strategies incorporating fatty acids (FAs) have become common in nutritional efforts to prevent malnutrition in some countries [2]. Results from trials of nutrient supplements containing FAs suggest that provision of FAs improves growth of infants and children [3, 4]. However, these lipid supplementation strategies are currently uncommon in Tanzania.

FAs are pivotal for the growth and development of humans. In addition to providing energy, FAs have several physiological functions. FAs have important structural and signaling roles in cell membranes as well as being important for vision, skin integrity, wound healing, heart health, cognition, and immune responses [5]. Linoleic (LA) and alpha-linolenic acid (ALA) are essential fatty acids (EFAs) because humans lack the Δ12- and Δ15-desaturase enzymes necessary for *de novo* synthesis of these FAs [6, 7]. Therefore, these FAs must be supplied through diet. When inadequate amounts of LA and ALA are consumed, dermatitis, hair loss, platelet deficiency and cognitive impairment ensue [8–10]. LA, an n-6 FA, and ALA, an n-3 FA, are metabolized *de novo* to longer-chain FAs by desaturases (i.e., Δ5- and Δ6-desaturase) and elongases [11]. These enzymes preferentially metabolize n-3 FAs and next prefer n-6 FAs over n-9 FAs [12]. When dietary intake of EFA is low, n-9 FAs are elongated and desaturated to form Mead acid [13]. Thus, in situations where individuals do not consume sufficient amounts of the EFAs, the non-essential n-9 FA oleic acid (C18:1n9) is converted to Mead acid (C20:3n9) [14]. Mead acid is incorporated in phospholipids, cholesterol esters, and triglycerides, as well as being found as a non-esterified free FA [15, 16]. EFA deficiency (EFAD) leads to higher levels of Mead acid and an elevated triene to tetraene ratio (the T/T ratio), which is defined as the ratio of Mead acid to arachidonic acid (ARA) [17–19]. Historically, a T/T ratio > 0.02 in plasma samples defines EFAD [17, 18]. Mead acid [13] levels above 0.4% [20] in red blood cells (RBCs) and 0.21% [18] in plasma have also been used to define EFAD.

In rural areas in Tanzania where malnutrition is highly prevalent, total fat intake is low [21]. This low fat intake increases the likelihood of EFAD in infants and young children. Dietary sources of EFAs include oils and seeds. Sources of LA include sunflower, corn, peanut, soybean oil, and canola oil, while flaxseed, walnut, and soy are rich sources of ALA [22]. Salmon, trout, eggs, poultry with skin, and whole grains also contain EFA [22, 23]. Young children in some areas of Tanzania either do not typically consume or do not consume sufficient quantities of these foods for them to be good sources of EFA. In particular, in the Rudewa Mbuyuni village, Kilosa district of Tanzania, a majority of the families consume two meals per day. A typical meal includes a large portion of carbohydrates (maize, cassava, and rice) served with sauces made from pulses and vegetables. Children between 2 and 6 years of age consume two meals and sometimes one snack if available. These children also consume between 200-300ml of a grain-based porridge per day. Thus, inadequate EFA intake may be partially responsible for the prevalence of growth stunting in Tanzanian infants and children.

Although there are FA supplementation studies in other developing countries, few report blood or plasma FA levels [24]. Additionally, levels of EFA intake have not been measured in children in Tanzania. Furthermore, blood FA levels in populations of children from developing countries, specifically Tanzania, are mostly unknown [24, 25] with some exceptions [25–32]. Given the importance of EFAs, there is a need to assess whole blood FA levels and their relationship to growth in developing countries such as Tanzania [24, 33]. Therefore, we assessed FA levels in 2-to-6 year old Tanzanian children to determine if some FA levels are associated with growth. We hypothesized that whole blood EFA levels in Tanzanian children would positively correlate with the growth parameters: weight-for-age (WAZ), weight-for-height (WHZ), height-for-age (HAZ) and BMI-for-age (BAZ) *z*-scores.

## Subjects and Methods

### Study setting [34]

The study was conducted in Rudewa Mbuyuni village, Kilosa district of Tanzania. Kilosa district covers a total area of 14,245 km^2^ of which, 5367 km^2^ are suitable for agriculture. Kilosa district is characterized by a dry tropical climate of semi-arid type. Rudewa Mbuyuni consists of five hamlets and about 820 households. The major economic activities in the village are crop farming and livestock keeping. Most villagers are subsistence farmers growing sisal, cotton, paddy, maize, sorghum, pearl millet, sunflower, simsim, cowpeas, pigeon peas, bananas, coconuts, tomatoes, pumpkins, and sweet potatoes. Increasing numbers of villagers are micro-scale traders. Most households keep domestic animals such as cattle, goats, sheep, donkeys, chickens, and ducks. About 98% of the households in Rudewa Mbuyuni have access to drinkable water sources. About 40% of houses in the village are roofed with galvanized iron sheets, while the rest are roofed with thatched grasses. Only half of the adult population has attained primary education and can read and write. Common diseases in the district include malaria, acute respiratory infections, diarrhea, anemia, parasitemia, and skin infections. In the village there is a dispensary, which offers health services. As per the national guideline, antenatal services are given to pregnant mothers. Children below five years old attend a growth-monitoring clinic once a month, and the children are de-wormed with albendazole and given vitamin A supplements twice a year (June and December). Most young children eat uji, a cornmeal-based porridge, in addition to consuming family meals.

### Sample size and subjects

Children (n = 334) between 2–6 years of age residing in Rudewa Mbuyuni village participated in the study. Power analysis based on an estimated medium effect size of 0.4 indicated that 242 participants would yield a power of 80%. We enrolled 334 participants, which raised our power to 90%. All households with children between the ages of 2–6 years were identified and invited to participate in the study. Children who were sick or hospitalized at the time of data collection as well as those who were legally declared intellectually disabled were excluded from the study. Consent was given by the parent or caregiver of the participating child. This consent was verbal because the majority of adults in this village are illiterate. A script of the written consent form that explained the objective of the study was read to the parent/caregiver. They were assured that participation was voluntary and confidential, and that their information would remain anonymous. Parents or caregivers then gave their verbal assent in Swahili. Swahili is the official national language and is used for instruction in primary school education in Tanzania. This study observed all the ethical standards of and was approved by the Institutional Review Board at Michigan State University (IRB#13–700) and the Tanzanian National Institute for Medical Research (NIMR/HQ/R.8a/Vol. IX/1189). Data were collected from December 2013 to August 2014.

### Anthropometric measurements

Height was measured to the nearest 0.1cm with a stadiometer (Shorr Productions, Perspective Enterprises, Portage, Missouri). Weight was measured using a digital bathroom scale to the nearest 0.1kg (A SECA, Vogel & Haike, Hamburg, Germany). Measurements were repeated, and the average of the two measurements was used. Date of birth of the child was recorded from the reproductive and child health clinic (RCH) card, and mother’s recall was used for those who did not have the RCH card. Sex of the child was also recorded.

### Blood measurements

A procedure similar to that used previously was utilized [35–40]. A drop of blood was obtained by puncturing the tip of the middle finger using sterile, single-use lancets [41]. This procedure was relatively painless. The first drop of blood was wiped away with a dry pad. Then, approximately 30μl of capillary blood was collected and applied to a dried blood spot (DBS) card (OxyStop) that had been pre-treated with an antioxidant cocktail composed of butylated hydroxyanisole, alpha-tocopherol and tertiary-butylhydroquinone [39, 41]. The cards were then stored in a dry and cool environment. The DBS cards were shipped to the USA for FA analysis at OmegaQuant Analytics, LLC (Sioux Falls, SD). The average time between sample collection and arrival at the US lab was 8 ± 5 days. After arrival in the US lab, the samples were analyzed as previously described [40, 42, 43]. Briefly, a punch from the DBS card was combined with the derivatizing reagent [boron trifluoride in methanol (14%), toluene, and methanol (35:30:35 parts)], shaken and heated at 100°C for 45 minutes. After cooling, 40 parts of both hexane and distilled water were added. After briefly vortexing, the samples were spun to separate layers and an aliquot of the hexane layer that contained the FA methyl esters was extracted. FA analysis was performed as previously described [44–46]. Unless otherwise stated, whole blood FA proportions are expressed as a percent of total identified FAs. Additional drops of blood from the same puncture site were used to assess hemoglobin (Hb) concentration and malaria status. An HemoCue photometer (HemoCue AB, Angelholm, Sweden) was used to measure hemoglobin (Hb) concentration. Malaria status was measured using a rapid test kit (Premier Medical Co. Ltd., India) and confirmed by blood smear.

### Data reduction and statistical analyses

The weight, height, date of birth and sex data were entered into WHO Anthro [47] and WHO AnthroPlus [48] to calculate *z*-scores. WHZ are missing for the 63 children who were older than 5 years of age. Data from those participants were excluded from analyses including WHZ. The WHO standard population and definitions of moderate and severe stunting, wasting, and underweight were applied to the data [49]. Each *z*-score indicates how many standard deviations from expected, as described by the WHO standard population, the individual in question falls on the basis of her anthropometric measurements. Stunted children have low HAZ. Wasted children have low WHZ. Underweight children have low WAZ.

Basic descriptive analyses were conducted to obtain means and frequencies. Pearson correlations were calculated for all continuous predictors and covariates. Since there was high collinearity between the various FAs, individual linear regression models were employed for each FA of interest. Regression models included the FA of interest, Hb concentration, and malaria status. T tests were conducted to identify differences in blood levels of FAs between boys and girls. SPSS version 22 (IBM Corporation, Armonk, NY) was used for these statistical analyses.

FA patterns were generated by principal components analysis (PCA). PCA reduces the number of variables and allows correlated variables to be assessed simultaneously. A linear transformation was performed to enable interpretation. In this case, varimax rotation was performed. Three factors were retained as determined by eigenvalues >1.2. The procedure assigns each person a score for each of the three factors that emerged from the data. Multiple linear regression using these factor scores as predictors was used to determine the relationships between these factors and the growth parameters. SAS version 9.4 (Cary, NC) was used for these statistical analyses.

## Results

### Subject Characteristics

In this study (**Table 1**), the mean age of children was 44.9 ± 14.8 months. There were more females (53.3%) than males (46.7%). The average height of participants was 94.3 ± 9.58 cm, and the average weight of participants was 13.9 ± 2.60 kg. Hb levels ranged from 6 g/dl to 15 g/dl, and 17% of the children tested positive for malaria. The mean HAZ was -1.52. The mean WAZ was -0.97. The mean WHZ was -0.086, and the mean BAZ was 0.03. No WHZ were calculated for the 63 children who were older than 5 years of age at the time of the study. The standard deviations of the HAZ, WAZ, and WHZ distributions were relatively constant and close to the expected value of 1.0 (range: 0.87–1.16). About a third (30.3%) of these children were stunted and 13% were underweight according to WHO criteria [49] (**Table 2**). Approximately, 1% were wasted or had a low BMI for their age.

**Table 1:** Demographic Characteristics of the Participants^1^.

	Mean ± SD			
Age groups, mos	18–36	36.1–48	48.1–60	> 60
	(n = 108)	(n = 96)	(n = 58)	(n = 72)
Age, mos	28.9 ± 3.95	41.2 ± 3.26	54.2 ± 3.75	66.6 ± 4.45
Sex, *n* (%) male	56 (51.9)	41 (42.7)	30 (51.7)	28 (38.9)
Height, cm	84.6 ± 4.10	93.1 ± 4.90	100 ± 5.35	106 ± 6.02
Weight, kg	11.5 ± 1.51	13.5 ± 1.67	15.2 ± 1.59	16.7 ± 2.18
HAZ	-1.71 ± 1.26	-1.48 ± 1.15	-1.40 ± 1.10	-1.52 ± 1.07
Hb, g/dL	9.91 ± 1.49	10.3 ± 1.43	10.7 ± 1.23	10.4 ± 1.62
+ malarial RDT, *n* (%)	17 (15.7)	11 (11.5)	14 (24.1)	14 (19.4)

^1^HAZ, height-for-age *z-*score; Hb, hemoglobin; mos, months; RDT, rapid detection test

**Table 2:** Nutrition and Growth Status of Children^1^.

	Based On	Severe	Moderate	Unaffected
		(< -3 SD)	(< -2 SD)	
Stunting	HAZ	9.9%	20.3%	69.9%
Malnutrition	BAZ	0%	1.2%	98.8%
Underweight	WAZ	2.1%	11%	86.9%
Wasting	WHZ	0%	1.5%	98.5%

^1^BAZ, BMI-for-age *z* score; HAZ, height-for-age *z* score; WAZ, weight-for-age *z* score; WHZ, weight-for-height *z* score. The WHO definitions of moderate and severe stunting, wasting, underweight and malnutrition were applied to the data [49].

### Fatty Acid Levels in Whole Blood

The mean proportions (mole percent) of selected FAs are shown in **Table 3**. Over one-fifth (23.1%) of these children had a whole blood T/T ratio greater than 0.02 while 16% of these children had whole blood Mead acid levels above 0.21%. The mean T/T ratio in this population was 0.016 ± 0.008. There was no significant difference between the T/T ratios for boys and girls (p = 0.137). Girls and boys had similar ARA levels, but boys had significantly higher Mead acid levels than girls (p = 0.04).

**Table 3:** Whole Blood Fatty Acid Proportions^1^.

Fatty Acid	Mean ± SD	Range
DHA	2.40 ± 0.66	0.88–4.34
ARA	9.16 ± 1.49	3.66–13.10
Myristic	1.00 ± 0.63	0.25–4.89
Palmitic	27.63 ± 1.92	22.63–33 82
Palmitelaidic	0.09 ± 0.07	0.002–0.49
Palmitoleic	1.61 ± 0.74	0.28–4.44
Stearic	10.39 ± 1.02	6.67–12.91
Elaidic	0.22 ± 0.21	0.06–3.52
Oleic	21.52 ± 2.98	14.21–32.74
Linoelaidic	0.33 ± 0.11	0.13–1.13
Linoleic	17.91 ± 2.79	11.48–31.44
Arachidic	0.23 ± 0.05	0.11–0.42
Glinolenic	0.28 ± 0.12	0.06–0.72
Eicosenoic	0.23 ± 0.07	0.09–0.49
Alpha-linolenic	0.37 ± 0.18	0.10–1.33
Eicosadienoic	0.24 ± 0.07	0.10–0.56
Behenic	0.56 ± 0.17	0.13–1.16
DGLA	1.56 ± 0.36	1.56–0.36
Lignoceric	0.60 ± 0.29	0.13 ± 1.62
EPA	0.38 ± 0.19	0.05–1.58
Nervonic	0.53 ± 0.25	0.08–1.50
Docosatetraenoic	1.15 ± 0.26	0.39–2.22
DPA n-6	0.65 ± 0.15	0.24–1.29
DPA n-3	0.79 ± 0.22	0.31–1.74
Mead	0.13 ± 0.06	0.02–0.39
Total n-3^2^	3.95 ± 0.89	1.82–6.25
Total n-6^3^	31.05 ± 3.51	21.58–40.63
Total n-9^4^	22.63 ± 2.93	14.99–33.43
Total SatFat^5^	40.42 ± 1.86	34.74–45.81
Total MUFA^6^	2.24 ± 0.72	0.74–5.42
Total PUFA^7^	35.00 ± 3.77	23.40–43.25

^1^Expressed as FA mol%; (n = 334). DHA, docosahexaenoic acid; EPA, eicosapentaenoic acid; T/T, triene-to-tetraene

^2^Total n-3 includes alpha-linolenic, EPA, docosapentaenoic n-3, and DHA.

^3^Total n-6 includes linoleic, linoelaidic, gamma-linolenic, eicosadienoic, di-homo-gamma-linolenic, arachidonic, docosatetraenoic, docosapentaenoic n-6.

^4^Total n-9 includes oleic, elaidic, eicosanoic, Mead, nervonic.

^5^Total saturated fat includes myristic, palmitic, stearic, arachidic, behenic, lignoceric.

^6^Total MUFA includes palmitoleic, palmitelaidic and nervonic

^7^Total PUFA included Total n-3 and Total n-6

### Correlations between Fatty Acids and Growth Parameters

Pearson correlations were calculated for all continuous predictors and covariates (**S1 Table**). HAZ was inversely correlated with T/T ratio (-0.154, p = 0.005) and whole blood Mead acid levels (-0.114, p = 0.037). Additionally, total n-9 and oleic acid levels were also inversely correlated with HAZ (p = 0.007). Whole blood LA levels were positively correlated with HAZ (0.157, p = 0.004). Also, total n-6 levels were positively correlated with HAZ (0.204, p<0.001). The BAZ and T/T ratio were positively correlated (0.123, p = 0.025) whereas an inverse correlation was observed between ARA (-0.13, p = 0.018), total n-6 (-0.127, p = 0.02) and BAZ. No significant associations were observed between WHZ and the blood FA levels except for ARA, which was negatively correlated WHZ (-0.159, p = 0.009). None of the FA correlated with WAZ.

### Regressions between Fatty Acids and Growth Parameters

Regression results for HAZ and some selected FAs are shown in **Table 4**. A strong negative relationship was observed between HAZ and the T/T ratio (p = 0.006). A similar strong negative relationship was observed for Mead acid, oleic acid and total n-9 FA with HAZ. A positive relationship was observed between HAZ and total n-6 FAs (p = 0.001) as well as LA (p = 0.008). On the other hand, Oleic acid, total n-9 FAs and the T/T ratio were positively associated with BAZ (**Table 5**). ARA and total n-6 FAs were negatively associated with BAZ (**Table 5**). No significant relationship was observed between any of the selected FAs and WAZ (**S2 Table**). A negative relationship was observed between WHZ and ARA as well as total n-6 FAs (p<0.043) (**S3 Table**). There was a positive relationship between WHZ and oleic acid as well as total n-9 FAs (p<0.044) (**S3 Table**). DHA, EPA, and total n-3 FAs were not associated with any growth parameters.

**Table 4:** Regression^1^ Results Between HAZ and Selected Fatty Acids.

Fatty Acid	B ± SE	T-value	p-value
Oleic	-0.052 ± .020	-2.503	**0.013**
Linoleic	0.061 ± .020	2.680	**0.008**
α-Linolenic	-0.248 ± .365	-0.680	0.497
Mead	-1.877 ± .888	-2.115	**0.035**
Arachidonic	0.070 ± .041	1.692	0.092
T/T ratio	-20.3 ± 7.37	-2.755	**0.006**
Total n-3^2^	0.058 ± .063	0.907	0.365
Total n-6^3^	0.064 ± .020	3.512	**0.001**
Total n-9^4^	-0.054 ±.020	-2.521	**0.012**
Total Saturated^5^	-0.071 ± 0.04	-1.977	**0.049**

^1^Model: HAZ = fatty acid + malaria status + hemoglobin concentration. HAZ, height-for-age *z* score; T/T, triene-to-tetraene

^2^Total n-3 includes alpha-linolenic, eicosapentaenoic, docosapentaenoic n-3, and docosahexaenoic.

^3^Total n-6 includes linoleic, linoelaidic, γ-linolenic, eicosadienoic, di-homo-gamma-linolenic, arachidonic, docosatetraenoic, docosapentaenoic n-6.

^4^Total n-9 includes oleic, elaidic, eicosanoic, Mead, nervonic.

^5^Total saturated fat includes myristic, palmitic, stearic, arachidic, behenic, lignoceric.

**Table 5:** Regression^1^ Results Between BAZ and Selected Fatty Acids.

Fatty Acid	B ± SE	T-value	p-value
Oleic	0.035 ± .016	2.14	**0.033**
Linoleic	-0.021 ± .018	-1.19	0.237
α-Linolenic	0.041 ± .284	0.145	0.885
Mead	1.09 ± .693	1.57	0.118
Arachidonic	-0.087 ± .032	-2.73	**0.007**
T/T ratio	13.26 ± 5.76	2.30	**0.022**
Total n-3^2^	-0.015 ± .049	-0.30	0.762
Total n-6^3^	-0.038 ± .014	-2.67	**0.008**
Total n-9^4^	0.053 ±.020	2.07	**0.039**
Total Saturated^5^	0.072 ± .028	1.32	0.189

^1^Model: BAZ = fatty acid + malaria status + hemoglobin concentration. BAZ, BMI-for-age *z-* score; T/T, triene-to-tetraene

^2^Total n-3 includes alpha-linolenic, eicosapentaenoic, docosapentaenoic n-3, and docosahexaenoic.

^3^Total n-6 includes linoleic, linoelaidic, gamma-linolenic, eicosadienoic, di-homo-gamma-linolenic, arachidonic, docosatetraenoic, docosapentaenoic n-6.

^4^Total n-9 includes oleic, elaidic, eicosanoic, Mead, nervonic.

^5^Total saturated fat includes myristic, palmitic, stearic, arachidic, behenic, lignoceric.

### Principal Component Analysis

When PCA was used to determine how combinations of these variables might be associated with growth, three factors emerged. The factor loading matrix is shown in **Table 6**. Multiple linear regression between these three factors and HAZ (p = 0.0453) revealed that Factor 1 (p = 0.024) and Factor 3 (p = 0.026) were significantly associated with stunting. Specifically, Factor 1 was positively associated with HAZ. Factor 3 was inversely associated with HAZ. In further multiple linear regression analyses, the overall models were not significant (WAZ (p = 0.700), WHZ (p = 0.432) or BAZ (p = 0.147)). However, Factor 1 was negatively associated with BAZ (p = 0.044).

**Table 6:** Factor Loading Matrix for Fatty Acids in the Whole Blood of 2–6 year old Tanzanian Children^1^.

	Factor 1:	Factor 2:	Factor 3:
Stearic	0.79	-0.04	-0.19
AA	0.89	0.05	-0.16
Docosatetraenoic	0.74	0.12	0.01
DPA n-6	0.65	0.08	0.12
DGLA	0.48	0.09	0.25
Linoleic	0.07	-0.00	-0.77
Eicosadienoic	0.39	0.24	-0.06
DPA n-3	0.60	0.05	0.34
DHA	0.56	0.13	-0.14
Lignoceric	0.27	0.86	0.02
Behenic	0.37	0.84	-0.04
Arachidic	0.11	0.87	-0.07
Palmitelaidic	0.14	0.34	-0.06
Eicosenoic	-0.07	0.30	0.07
Elaidic	-0.08	0.29	-0.15
Nervonic	0.31	0.82	0.04
Linoelaidic	-0.19	0.21	0.20
Palmitic	-0.61	-0.29	0.48
Myristic	-0.13	-0.22	0.29
Mead	-0.06	-0.09	0.66
Palmitoleic	-0.23	-0.30	0.75
Oleic	-0.79	-0.12	0.21
Gamma-linolenic	-0.03	-0.06	0.48
EPA	0.21	0.28	0.45
Alpha-linolenic	0.10	0.16	0.38

^1^The factor loading value indicates the correlation between the fatty acid and the factor. AA, arachidonic acid; DPA, docosapentaenoic acid; DGLA, dihomo-gamma-linolenic acid; DHA, docosahexaenoic acid; EPA, eicosapentaenoic acid

## Discussion

In summary, some blood FAs in Tanzanian children were associated with growth parameters. The n-6 FAs were inversely associated with stunting and positively associated with wasting. The n-9 FAs were positively associated with stunting and negatively associated with underweight. LA, an n-6 FA, was inversely associated with stunting. However, although it has been shown that red blood cell DHA levels positively correlate with plasma insulin-like growth factor-1 concentrations [50], neither ALA nor total n-3 were associated with any growth parameters. This lack of association is consistent with some [51, 52] but not all [52, 53] previous reports. Tanzanian children in this study had higher whole blood Mead acid levels compared to those previously reported for European children [54]. Further, about 16% of all participants had Mead acid levels above 0.21%, and 23% of all participants had a T/T ratio greater than 0.02 indicating potential deficiencies in EFA intake. Children with high whole blood levels of Mead acid and elevated T/T ratios were at risk of being stunted. This was evidenced by the inverse association of the T/T ratio and Mead acid with HAZ.

These data suggest that n-6 FAs are important for linear growth whereas n-9 FAs participate in the accumulation of overall body mass but may not play a role in linear growth. The linear growth-restriction observed in children with higher whole blood Mead acid and T/T ratios may explain the positive relationship between BAZ and Mead acid, as well as the positive relationship between BAZ and T/T ratio. It is plausible that a shorter child consuming the same number of calories as a taller child and gaining the same amount of body weight as a taller child would have a larger BMI. This could also explain why n-6 FAs were negatively associated with WHZ and BAZ despite being positively associated with HAZ; whereas n-9 FAs were negatively associated with HAZ but positively associated with WHZ and BAZ (**Table 7**).

**Table 7:** Summary of Significant Associations Between Fatty Acids and Growth Parameters.

	GROWTH PARAMETERS		
	HAZ	BAZ	WHZ
Oleic	⬇	⬆	⬆
Mead	⬇	---	---
ARA	---	⬇	⬇
Total n-9	⬇	⬆	⬆
Total n-6	⬆	⬇	⬇
T/T ratio	⬇	⬆	---

Two to six years of age is a period of rapid growth, and thus a time of increased need for nutrients. Therefore, in regions where diet quality is poor, children of these ages are likely to develop deficiencies of many nutrients, including FAs. The mixed ability of micronutrient supplementation to improve the growth of children in the developing world continues to be a source of confusion [55, 56]. Recently the provision of fat and/or energy has been emphasized in studies trying to optimize growth [57, 58]. There are limited data available in developing countries to support higher intakes of EFA as a way to improve growth [56, 59, 60]. The data that is available comes primarily from studies in which pregnant women, lactating women or infants receive FA supplements. Research in these populations suggests that an adequate supply of EFAs and n-6 FAs are required for normal in utero and early childhood growth [24, 61–66]. Others have suggested that EFAs are important for optimal linear growth [56]. In their study comparing the effectiveness of three micronutrient supplements, only one of which also contained FAs (LA and ALA only), the increased energy intake from the FAs did not explain the increase in linear growth for infants receiving the EFA-containing supplement [56]. The infants receiving the EFA supplement also tended to be more likely to be walking independently than those infants who received micronutrients alone. Furthermore, the infants in the EFA group had greater plasma ALA and lower plasma saturated fat levels, and it was this difference that explained the majority of the linear growth differences compared to the groups that received micronutrient supplements alone [25]. It’s not clear why blood n-6 FA levels were not observed to affect growth in this population. Although the data from human studies attempting to connect FA intake and linear growth is mixed, data from animal studies clearly indicate that n-6 FAs are essential for optimal growth, and there have been suggested mechanistic links.

Two potential mechanisms by which n-6 FAs may affect growth include 1) functioning as an important structural barrier to prevent energy loss and 2) being metabolized into molecules with specificity for receptors whose signal transduction pathways result in changes to linear growth. FAs provide energy and serve as building blocks for the membranes of cells and cellular compartments. Although there is some debate as to whether LA or ARA is the essential n-6 FA, in the two-to-six year old Tanzanian children, only LA was significantly positively associated with HAZ (**Table 4**). This may be because the n-6 EFA, LA, plays an important role in the structure of skin ceramides [67]. Because of its important role in skin ceramides, LA deficiency results in decreased water barrier and decreased thermal insulation leading to increased energy expenditure [68]. In an animal study, growth retardation was prevented when LA deficient animals were kept at 30°C and in humid conditions that minimized water evaporation from the skin [69]. This demonstrates that trans-epidermal water loss and accompanying heat loss can account for the main symptoms of LA deficiency including growth retardation, hyperphagia and hyperdipsia. These factors could contribute to the inefficient use of dietary calories during EFAD and lead to a reduced efficiency of growth [12, 70].

FAs are also signaling molecules. FAs can bind directly to receptors or stimulate cell signaling after they have been converted from their original structure to new metabolites [71]. Both the n-6 and n-3 classes of FAs are converted into eicosanoids [72]. However, the specific eicosanoid produced depends on the parent compound. Thus, the positive associations between n-6 FAs and growth may be related to the activity of n-6 FA metabolites. For instance, prostaglandin E_2_ (PGE2), a cyclooxygenase metabolite of the LA elongation product ARA [73] increases insulin-like growth factor-1 expression and increases calcium accretion in humans [74]. The n-6 FAs also contribute to bone accretion [75, 76] perhaps through inhibition of osteoclast formation, differentiation and activity [77]. Thus, n-6 FAs can stimulate linear growth of children. ARA can also be metabolized into endocannabinoids, molecules that play a pivotal role in stimulating appetite and controlling metabolism [78]. Therefore, in the absence of sufficient n-6 FAs, insufficient endocannabinoids may be synthesized, and growth could be compromised. Furthermore, conversion of ARA to its metabolites may reduce the ability to detect un-metabolized ARA in the whole blood. This could be why ARA was not associated with HAZ in the children of Rudewa Mbuyuni.

Because whole blood levels of certain FAs are reflective of dietary FA intake [20, 79, 80], the dietary customs of the children in Rudewa Mbuyuni are important factors with regard to understanding the results of this research. In Rudewa Mbuyuni the majority of children are breastfed up to two years (70.4%) as is the custom in most areas of Tanzania [81]. Human milk FA composition is directly influenced by diet [70]. Breastfed children typically consume sufficient EFA due to the presence of EFA in human milk [82]. Studies by Kuipers et al. [28, 83], conducted in three different areas in Tanzania, have shown that human milk FA composition is related to dietary consumption. Overall in Tanzania, the human milk FA composition is high in palmitic acid and low in DHA. However, it has previously been observed that food intake varies depending on location within Tanzania, with individuals living in rural and urban areas consuming different foods [84]. The FA composition of human milk from mothers in Rudewa Mbuyuni is unknown. Most breastfed children living in Rudewa Mbuyuni also eat a cereal-based porridge that is high in carbohydrates but is not nutrient dense [85]. By the time they are one year old, infants start to consume family meals. In Rudewa Mbuyuni, food patterns and habits are homogenous—families usually eat together, and meals are high in carbohydrates and low in fat [34]. A typical meal includes a large portion of carbohydrates (maize, cassava, and rice) served with sauces made from pulses and vegetables. These foods usually do not contain sufficient amounts of fat, nor sufficient amounts of EFAs. Additionally, poor feeding practices [86] and exposure to infectious diseases likely affect digestion and absorption leading to malabsorption and predisposing children to poor FA status. Thus, in Rudewa Mbuyuni, it is possible that dietary deficiency of EFAs is one of the factors contributing to the high whole blood Mead acid and T/T ratios and the corresponding poor growth. However, polymorphisms in FADS1 and FADS2, fatty acid desaturases responsible for elongating the EFA to ARA, EPA and DHA, have been associated with decreased height (anywhere from 0.1cm to 1.2cm) and decreased weight (around 2.2kg) in the Inuit as well as in some individuals of European ancestry [87]. In Africa, a fatty acid desaturase variants associated with increased production of DHA, ARA and EPA are prevalent [72, 88]. Thus, rapid conversion of LA to ARA could limit the amount of LA available for incorporation into skin ceramides, but may increase endocannabinoid levels. Additionally, dietary intake of other FAs affects desaturase and elongase enzyme activities [89–92] so intake of other dietary FAs, such as high levels of oleic acid [29, 90], could be responsible for the EFA levels observed in the current study. Unfortunately, it was outside the scope of the present study to assess FADS genotype or dietary intake. Further research is needed to determine the specific cause(s) of the FA blood levels observed in the children of Rudewa Mbuyuni.

Historically, FA levels in plasma are used to determine EFAD. As mentioned, the T/T ratio is the ratio of Mead acid to ARA. A T/T ratio > 0.02 in plasma samples defines EFAD [17, 18]. Mead acid [13, 93] levels above 0.4% [20] in red blood cells (RBCs) and 0.21% [18] in plasma have also been used to define EFAD. Mead acid is incorporated in all blood lipid sub-classes but mainly in the triacylglycerol and phospholipid compartments [40, 41]. Mead acid is less abundant in cholesterol esters and the non-esterified lipid fraction [16]. Studies assessing EFAD have shown no significant difference when assessing Mead acid in either phospholipids or triglycerides [15]. The dried blood spot method utilized in the current study analyzes whole blood samples in which the FA proportions are roughly derived 50:50 from red blood cells and plasma [94]. In two to six year old children, particularly those with anemia, this 50:50 estimate may be high as children typically have hematocrit values between 37 and 40% [95]. Since the current analysis investigated Mead acid in whole blood, the levels reported reflect Mead acid levels in several lipid fractions. The precise T/T ratio associated with EFAD in whole blood remains to be determined. We have observed a strong correlation between ARA in whole blood and ARA in plasma (r^2^ = 0.89, n = 50; WSH unpublished data), and others have reported the same with most correlation coefficients falling between 0.8 and 0.97 for PUFA [96]. Although we have not measured Mead acid in whole blood and plasma from the same individuals, it is likely Mead acid detection would be similar to that for ARA. Thus, the T/T ratio in plasma and whole blood likely would be similar for any one individual. Therefore, it is feasible that a cut point of >0.02 for the T/T ratio in whole blood could be used as a biomarker of EFAD.

Several limitations of this research must be acknowledged. During this study, blood samples were collected throughout the day and no fasting was required. This might be expected to increase variability in the whole blood FA measurements; however, in this study setting the differences are likely to be small considering that children from the village consume relatively similar and low-fat meals compared to children in other settings. We did not collect any measurements of body fat, such as mid-upper arm circumference, bioimpedance, underwater weighing or BOD-POD (Cosmed, Chicago, IL) data. Others have reported that BMI is correlated with these other direct measures of body fat [97–99]. Deficiencies of some nutrients may interfere with FA metabolism and affect whole blood FA levels. Furthermore, deficiencies of some nutrients can also contribute to poor growth. In this study we did not assess adequacy of other nutrients, such as zinc or protein, which are potentially inadequate in the diets of the children. Therefore, we do not know if children with high Mead acid and T/T ratios also had other nutritional deficiencies. Since this was a cross-sectional study, all reported associations are correlative rather than causative. This study was limited to participants from one village in Tanzania. For this reason and because it has previously been observed that food intake differs between Tanzanians living in rural and urban areas consuming different foods [84], these results are not generalizable to the entire Tanzanian population of children. No socio-economic data was collected from the children’s parents/caregivers; however, the population is relatively homogeneous as explained by Ntwenya et al. [34]. For these reasons, the results should be interpreted with caution

There were several strengths of this study. Among these strengths is the inclusion of biomarkers rather than reliance on food intake questionnaires to define FA exposure. Whole blood FA levels in 2-to-6 year old Tanzanian children have not previously been measured. In fact, the current study is one of a limited number of studies [25, 30, 31] to assess the relationship between growth parameters and blood FA levels directly instead of using dietary intake or supplementation regimens to determine FA status. Other strengths include assessments by well-trained anthropometrists and the recruitment of nearly all eligible children of the appropriate ages in the village. Because a large number of children participated, the study was well-powered to detect differences in the parameters of interest. Further, the use of an oxidation-inhibiting collection card (Omega Quant Analytics, LLC (Sioux Falls, SD)) enabled samples to be shipped to the United States for direct measurements of whole blood FA levels. This system has previously been used, and FAs were determined to be stable when collected and analyzed by these methods [36–38, 40]. Since it is difficult and costly to separate RBCs and plasma from whole blood during field work in developing countries and then isolate phospholipids from different blood fractions (i.e., RBC and plasma), the current method of DBS analysis increases the feasibility of assessing EFAD in remote areas where EFAD is more prevalent.

## Conclusion

This study assessed whole blood levels of EFAs and the association between whole blood FA levels and growth parameters in children 2–6 years of age. EFA levels in 2-to-6 year old Tanzanian children were low as demonstrated by high levels of Mead acid and large T/T ratios, both markers of EFAD. In this population, levels of certain FAs in blood were associated with growth parameters. Most notably, higher n-6 FA levels were associated with greater linear growth whereas higher n-9 FA levels were associated with impaired linear growth. The inadequate levels of FAs in this population may not only be associated with impaired growth, but may also be associated with impaired immunity or poor cognitive development. Thus, future studies of nutritional interventions for Tanzanian children should consider providing FAs in addition to sufficient calories, protein and micronutrients. Additionally, these studies should encourage the incorporation of locally available FA-rich seeds and oils into meals and snacks for infants and children. Finally, the development of simple instrumentation that can be used in the field to analyze FAs and FA metabolites in whole blood would be useful for future studies. In sum, this study sets the foundation for future studies assessing the relationship between whole blood FA composition and growth in children residing in other areas of Tanzania, ideally over their entire life course.

## Supporting Information

S1 Table Correlations between Variables^1^. (DOCX)

S2 Table Regression^1^ Results Between WAZ and Selected Fatty Acids. (DOCX)

S3 Table Regression^1^ Results Between WHZ and Selected Fatty Acids. (DOCX)

## Abbreviations

ARA: arachidonic acid
ALA: alpha-linolenic acid
BAZ: BMI-for-age
BMI: body mass index
DBS: dried blood spot
DGLA: Dihomo-γ-linolenic acid
DHA: docosahexaenoic acid
EFA(s): essential fatty acid(s)
EFAD: essential fatty acid deficiency/deficient
EPA: eicosapentaenoic acid
FA(s): fatty acid(s)
HAZ: height-for-age z-score
Hb: hemoglobin
LA: linoleic acid
LCPUFA: long chain poly-unsaturated fatty acids
PCA: principle components analysis
RBC: red blood cell
RCH: Reproductive and Child Health Clinic
RDT: rapid diagnostic test
T/T: triene to tetraene ratio
WAZ: weight-for-age *z*-score
WHZ: weight-for-height *z*-score
