# Supplementary material for: Whole Blood Levels of the n-6 Essential Fatty Acid Linoleic Acid Are Inversely Associated with Stunting in 2-to-6 Year Old Tanzanian Children: A Cross-Sectional Study
Source: PLoS One. 2016 May 3;11(5):e0154715. doi: 10.1371/journal.pone.0154715 (PMC4854382; doi:10.1371/journal.pone.0154715)
Supplement: S1 Table — (DOCX) [file pone.0154715.s001.docx]

S1 Table. Correlations between Variables^1^

|  | Hb | Oleic | Linoleic | α-Linolenic | Nervonic | Mead | ARA | T/T Ratio | Total n-6 | Total n-9 | HAZ | WAZ | BAZ | WHZ |
| --- | --- | --- | --- | --- | --- | --- | --- | --- | --- | --- | --- | --- | --- | --- |
| Age | **0.166** | -0.077 | -0.044 | 0.102 | 0.038 | -0.067 | 0.081 | -0.085 | 0.038 | -0.072 | 0.097 | -0.075 | **-0.224** | -0.075 |
|  | **0.002** | 0.162 | 0.422 | 0.062 | 0.490 | 0.224 | 0.141 | 0.120 | 0.490 | 0.187 | 0.079 | 0.170 | **<0.001** | 0.215 |
| Hb | 1.00 | **-0.189** | 0.103 | -0.104 | 0.084 | 0.019 | **0.216** | -0.054 | **0.205** | **-0.190** | 0.050 | 0.088 | 0.069 | 0.039 |
|  |  | **0.001** | 0.060 | 0.058 | 0.124 | 0.734 | **<0.001** | 0.322 | **<0.001** | **<0.001** | 0.366 | 0.110 | 0.206 | 0.524 |
| Oleic |  | 1.00 | **-0.445** | -0.061 | **-0.302** | **0.183** | **-0.733** | **0.403** | **-0.780** | **0.992** | **-0.147** | -0.059 | 0.100 | 0.117 |
|  |  |  | **<0.001** | 0.265 | **<0.001** | **0.001** | **<0.001** | **<0.001** | **<0.001** | **<0.001** | **0.007** | 0.282 | 0.067 | 0.054 |
| Linoleic |  |  | 1.00 | **-0.205** | -0.077 | **-0.414** | 0.100 | **-0.414** | **0.810** | **-0.466** | **0.157** | 0.102 | -0.058 | -0.032 |
|  |  |  |  | **<0.001** | 0.162 | **<0.001** | 0.067 | **<0.001** | **<0.001** | **<0.001** | **0.004** | 0.062 | 0.291 | 0.594 |
| α-Linolenic |  |  |  | 1.00 | **0.165** | -0.004 | 0.008 | 0.003 | -0.134 | -0.038 | -0.043 | -0.045 | -0.004 | 0.020 |
|  |  |  |  |  | **0.002** | 0.943 | 0.879 | 0.950 | 0.014 | 0.490 | 0.431 | 0.414 | 0.941 | 0.742 |
| Nervonic |  |  |  |  | 1.00 | -0.065 | **0.310** | **-0.155** | 0.136 | **-0.199** | 0.011 | -0.017 | -0.040 | -0.056 |
|  |  |  |  |  |  | 0.239 | **0.000** | **0.005** | 0.013 | **<0.001** | 0.837 | 0.753 | 0.463 | 0.359 |
| Mead |  |  |  |  |  | 1.00 | **-0.148** | **0.933** | **-0.371** | **0.197** | **-0.114** | -0.043 | 0.089 | 0.071 |
|  |  |  |  |  |  |  | **0.007** | **<0.001** | **<0.001** | **<0.001** | **0.037** | 0.435 | 0.105 | 0.247 |
| ARA |  |  |  |  |  |  | 1.00 | **-0.441** | **0.641** | **-0.723** | 0.107 | 0.003 | **-0.130** | **-0.159** |
|  |  |  |  |  |  |  |  | **<0.001** | **<0.001** | **<0.001** | 0.052 | 0.960 | **0.018** | **0.009** |
| T/T Ratio |  |  |  |  |  |  |  | 1.00 | **-0.542** | **0.411** | **-0.154** | -0.054 | **0.123** | 0.109 |
|  |  |  |  |  |  |  |  |  | **<0.001** | **<0.001** | **0.005** | 0.324 | **0.025** | 0.072 |
| Total n-6^2^ |  |  |  |  |  |  |  |  | 1.00 | **-0.789** | **0.204** | 0.093 | **-0.127** | -0.112 |
|  |  |  |  |  |  |  |  |  |  | **<0.001** | **<0.001** | 0.089 | **0.020** | 0.066 |
| Total n-9^3^ |  |  |  |  |  |  |  |  |  | 1.00 | **-0.148** | -0.062 | 0.096 | 0.115 |
|  |  |  |  |  |  |  |  |  |  |  | **0.007** | 0.255 | 0.079 | 0.059 |
| HAZ |  |  |  |  |  |  |  |  |  |  | 1.00 | **0.758** | **-0.142** | 0.048 |
|  |  |  |  |  |  |  |  |  |  |  |  | **<0.001** | **0.009** | 0.427 |
| WAZ |  |  |  |  |  |  |  |  |  |  |  | 1.00 | **0.535** | **0.675** |
|  |  |  |  |  |  |  |  |  |  |  |  |  | **<0.001** | **<0.001** |
| BAZ |  |  |  |  |  |  |  |  |  |  |  |  | 1.00 | **0.976** |
|  |  |  |  |  |  |  |  |  |  |  |  |  |  | **<0.001** |

^1^Top number is the Pearson’s correlation coefficient. Bottom number is the p-value. All significant associations (p<0.05) are in bold. ARA, arachidonic acid; BAZ, BMI-for-age *z* score; HAZ, height-for-age *z* score; Hb, hemoglobin; T/T, triene-to-tetraene; WAZ, weight-for-age *z* score; WHZ, weight-for-height *z* score

^2^Total n-6 includes LA, linoelaidic, γ-linolenic, eicosadienoic, di-homo-gamma-linolenic, arachidonic, docosatetraenoic, docosapentaenoic n-6.

^3^Total n-9 includes oleic, elaidic, eicosanoic, Mead, nervonic.
