# Supplementary material for: Whole Blood Levels of the n-6 Essential Fatty Acid Linoleic Acid Are Inversely Associated with Stunting in 2-to-6 Year Old Tanzanian Children: A Cross-Sectional Study
Source: PLoS One. 2016 May 3;11(5):e0154715. doi: 10.1371/journal.pone.0154715 (PMC4854382; doi:10.1371/journal.pone.0154715)
Supplement: S2 Table — (DOCX) [file pone.0154715.s002.docx]

S2 Table. Regression^1^ Results Between WAZ and Selected Fatty Acids

| Fatty Acid | B ± SE | T-value | p-value |
| --- | --- | --- | --- |
| Oleic | -0.012 ±.017 | -0.716 | 0.475 |
| Linoleic | 0.030 ± .018 | 1.622 | 0.235 |
| α-Linolenic | -0.172 ± .292 | -0.591 | 0.555 |
| Mead | -0.595 ± .713 | -0.835 | 0.405 |
| Arachidonic | -0.013 ± .033 | -0.392 | 0.695 |
| T/T ratio | -5.304 ± 5.95 | -0.891 | 0.374 |
| Total n-3^2^ | 0.025 ± .051 | 0.496 | 0.620 |
| Total n-6^3^ | 0.019 ± .015 | 1.283 | 0.200 |
| Total n-9^4^ | -0.013 ± .017 | -0.772 | 0.441 |
| Total Saturated^5^ | -0.025 ± .029 | -0.865 | 0.387 |

^1^Model: WAZ = fatty acid + malaria status + hemoglobin concentration. WAZ, weight-for-age *z* score; T/T, triene-to-tetraene

^2^Total n-3 includes alpha-linolenic, eicosapentaenoic, docosapentaenoic n-3, and docosahexaenoic.

^3^Total n-6 includes linoleic, linoelaidic, γ-linolenic, eicosadienoic, di-homo-gamma-linolenic, arachidonic, docosatetraenoic, docosapentaenoic n-6.

^4^Total n-9 includes oleic, elaidic, eicosanoic, Mead, nervonic.

^5^Total saturated fat includes myristic, palmitic, stearic, arachidic, behenic, lignoceric.
