# Supplementary material for: Whole Blood Levels of the n-6 Essential Fatty Acid Linoleic Acid Are Inversely Associated with Stunting in 2-to-6 Year Old Tanzanian Children: A Cross-Sectional Study
Source: PLoS One. 2016 May 3;11(5):e0154715. doi: 10.1371/journal.pone.0154715 (PMC4854382; doi:10.1371/journal.pone.0154715)
Supplement: S3 Table — (DOCX) [file pone.0154715.s003.docx]

S3 Table. Regression^1^ Results Between WHZ and Selected Fatty Acids

| Fatty Acid | B ± SE | T-value | p-value |
| --- | --- | --- | --- |
| Oleic | 0.037 ± .018 | 2.081 | **0.038** |
| Linoleic | -0.012 ± .019 | -0.648 | 0.518 |
| α-Linolenic | 0.137 ± .302 | 0.453 | 0.651 |
| Mead | 0.801 ± .709 | 1.129 | 0.260 |
| Arachidonic | -0.101 ± .036 | -2.847 | **0.005** |
| T/T ratio | 10.86 ± 5.95 | 1.826 | 0.069 |
| Total n-3^2^ | 0.006 ± 0.05 | 0.106 | 0.916 |
| Total n-6^3^ | -0.035 ± .015 | -2.044 | **0.042** |
| Total n-9^4^ | 0.037 ± .018 | 2.036 | **0.043** |
| Total Saturated^5^ | 0.003 ± .03 | 0.107 | 0.915 |

^1^Model: WHZ = fatty acid + malaria status + hemoglobin concentration. WHZ, weight-for-height *z* score; T/T, triene-to-tetraene

^2^Total n-3 includes ALA, EPA, DPA n-3, and DHA.

^3^Total n-6 includes LA, linoelaidic, γ-linolenic, eicosadienoic, DGLA, ARA, docosatetraenoic, DPA n-6.

^4^Total n-9 includes oleic, elaidic, eicosanoic, Mead, nervonic.

^5^Total saturated fat includes myristic, palmitic, stearic, arachidic, behenic, lignoceric.
